# The Effect of COVID-19 Severity, Associated Serum Autoantibodies and Time Interval after the Disease on the Outcomes of Fresh Oocyte ART Cycles in Non-Vaccinated Patients

**DOI:** 10.3390/jcm12134370

**Published:** 2023-06-29

**Authors:** Nataliya V. Dolgushina, Irina V. Menzhinskaya, Daria M. Ermakova, Natalia A. Frankevich, Valentina V. Vtorushina, Gennady T. Sukhikh

**Affiliations:** 1 National Medical Research Center for Obstetrics, Gynecology and Perinatology Named after Academician V.I. Kulakov of Ministry of Healthcare of the Russian Federation, 117997 Moscow, Russia; n_dolgushina@oparina4.ru (N.V.D.); i_menzinskaya@oparina4.ru (I.V.M.); n_frankevich@oparina4.ru (N.A.F.); v_vtorushina@oparina4.ru (V.V.V.); g_sukhikh@oparina4.ru (G.T.S.); 2 Department of Obstetrics, Gynecology, Perinatology and Reproductology, Institute of Professional Education, Federal State Autonomous Educational Institution of Higher Education the First Moscow State Medical University Named after I.M. Sechenov of Ministry of Health of the Russian Federation (Sechenov University), 119048 Moscow, Russia

**Keywords:** COVID-19, SARS-CoV-2, autoantibodies, infertility, assisted reproductive technology, reproductive outcomes

## Abstract

It is assumed that SARS-CoV-2- and COVID-19-associated autoimmune processes may affect the outcomes of assisted reproductive technology (ART) cycles. This observational prospective study included 240 infertile patients: 105 patients had no history of COVID-19 (group 1) and 135 patients had experienced COVID-19 (group 2) in a mild (n = 85) or moderate (n = 50) form less than 12 months prior to oocyte retrieval. Using ELISAs, the profiles of their serum autoantibodies were determined, including antiphospholipid antibodies and antibodies to nuclear and thyroid antigens. The parameters of oogenesis and embryogenesis, as well as the pregnancy and childbirth rates, did not differ between groups 1 and 2, and also between the subgroups with different severities of COVID-19. However, when oocyte retrieval was performed less than 180 days after COVID-19, a higher proportion of poor-quality blastocysts was obtained (*p* = 0.006). A high risk of early miscarriage was found in the patients with moderate COVID-19. In group 2, IgG antibodies to annexin V, phosphatidylethanolamine (PE), and TSHr were detected more often than in group 1 (*p* = 0.035; *p* = 0.028; and *p* = 0.033, respectively), and a weak inverse correlation was revealed between anti-PE IgG and the number of oocytes and zygotes obtained. The results of the study suggest a possible adverse effect of COVID-19 and its associated autoantibodies on the outcomes of fresh oocyte ART cycles and early pregnancy, which depends on the severity of COVID-19 and the time interval after the disease.

## 1. Introduction

The COVID-19 (COronaVIrus Disease 2019) pandemic, caused by the global spread of the novel, devastating coronavirus SARS-CoV-2 (Severe Acute Respiratory Syndrome-related CoronaVirus 2), was declared by the World Health Organization in March 2020. Over the past 3 years, more than 750 million cases of the disease have been registered globally and more than 6.8 million deaths have occurred. SARS-CoV-2 infection is able to trigger various immunopathological mechanisms in humans: inflammation, apoptosis, endothelial dysfunction, cytokine storm, and complement and coagulation hyperactivation [1]. SARS-CoV-2 is an enveloped, non-segmented single-stranded RNA virus of the genus Betacoronavirus [2]. Together, the nucleocapsid protein (N) and RNA form a large ribonucleoprotein complex, which is covered with lipids and other structural proteins: membrane protein (M), viral envelope protein (E), and spike protein (S) [3].

The entry of viral particles into human cells is mediated by the interaction of S protein with angiotensin-converting enzyme 2 (ACE2) on the cell membrane surface [4]. ACE2 is the main SARS-CoV-2 receptor and is widely presented in various tissues [5]. A high level of ACE2 expression in women has been noted in the tissues of the ovaries, uterus, and vagina [6]. An expression of the transmembrane serine protease 2 (TMPRSS2), which is necessary for the cleavage of the S protein on the cell surface and the subsequent fusion of the viral and cell membranes [4], has been found in the cells of the endometrium, placenta, ovaries, and fallopian tubes [6]. Recently, a co-expression of ACE2 and TMPRSS2 has been revealed in embryonic trophectoderm cells at the blastocyst stage [7].

Importantly, ACE2 is also a key component of the renin–angiotensin system (RAS) that affects reproductive processes in women [8]. Changes in ACE2 activity can lead to a dysregulation of the RAS and, as a consequence, to impaired folliculogenesis, ovulation, and corpus luteum function. The dysregulation of tissue RAS was observed in patients with COVID-19 and was assumed to be the main mechanism for the development of severe forms of the disease [9].

There are a number of publications that have discussed the possible adverse effects of COVID-19 on women’s reproductive health and fertility [10,11,12] and the limited clinical data on the outcomes of assisted reproductive technology (ART) cycles following COVID-19 [13,14]. It is plausible that the virus may affect ovarian and endometrial function. Isolated cases of infertility and premature ovarian failure have been reported in young patients with a history of COVID-19 [11,12]. In addition, it has been shown that pregnant women infected with SARS-CoV-2 had a 1.7-fold increased risk of early spontaneous miscarriage [15]. Moreover, the age of patients and the severity of COVID-19 may contribute to the development of pregnancy complications [16].

A significant decrease in the proportion of high-quality embryos in ART cycles after COVID-19 has been shown in a small case series (nine couples), with an interval from recovery to ART treatment between 8 and 92 days [17]. It is hypothesized that exposure to SARS-CoV-2 infection, which induces systemic inflammation, may reduce the quality of the developing embryos. In another study, there was a slight decrease in the rate of blastocyst formation in the post-COVID-19 group, with an interval between infection and ART treatment from 4 to 10 months, but there was no evidence that asymptomatic or mild SARS-CoV-2 infection could adversely affect female fertility, embryologic parameters, or clinical ART outcomes [18]. A decrease in the rate of blastocyst formation may be associated with the negative effect of oxidative stress on the quality of oocytes [19], as well as with a possible additional interaction between the virus and the developing embryo [13]. In a recent retrospective study, a negative effect of COVID-19 on oocyte yield was observed in patients who had had oocyte retrieval more than 180 days after infection, although the number of cases in this subgroup was small (n = 15) [20]. There are no data on the minimum required delay in ART treatment after COVID-19, if any, to ensure optimal outcomes [14].

In addition to the possible direct harmful effects of SARS-CoV-2 on reproductive structures, including oocytes and the embryo, or indirect effects through the RAS, the involvement of autoimmune mechanisms may also contribute to reproductive disorders in post-COVID-19 patients and affect the outcomes of assisted reproductive technologies ART cycles [21]. SARS-CoV-2 shares similarities with other viruses such as parvovirus B19, the Epstein–Barr virus, and cytomegalovirus, which are environmental triggers of autoimmunity in genetically predisposed people [22]. SARS-CoV-2 induces the production of autoantibodies in patients with severe COVID-19, causes a strong type I interferon response in some patients, and triggers autoimmune disorders in susceptible people [23]. Moreover, S protein contains superantigen motifs, and viral proteins have peptide sequences homologous to human protein fragments, which are widely distributed in various tissues of the human body; it is assumed that these proteins may be involved in the development of multiple organ damage through the mechanism of molecular mimicry [24]. In addition, patients with severe COVID-19 have elevated levels of extrafollicular B-lymphocytes and plasmatic cells, which develop from naive B-cells along an extrafollicular pathway lacking some tolerance checkpoints; this contributes to the triggering of autoimmune processes [25].

Post-COVID-19 autoimmune diseases have been reported recently, i.e., Graves’ disease and autoimmune hyperthyroidism, which are associated with the production of antithyroid antibodies; systemic lupus erythematosus (SLE), which is associated with the production of antinuclear antibodies (ANA) and antibodies to double-stranded DNA (anti-dsDNA); and antiphospholipid syndrome (APS) [26].

In connection with the above, studies on the impact of SARS-CoV-2, as well as the autoimmune processes associated with COVID-19, on women’s reproductive health and the outcomes of ART cycles are of great importance.

The aim of present study was to assess the impact of COVID-19 of varying severities and its associated autoantibodies on the reproductive outcomes of ART cycles, with fresh oocytes being retrieved at different time intervals after the disease.

## 2. Materials and Methods

This observational prospective study included 240 women who applied for infertility treatment using ART programs at the National Medical Research Center for Obstetrics, Gynecology and Perinatology, named after Academician V.I. Kulakov, from September 2020 to December 2021. The inclusion criteria were: an age of 18–40 years; a normal ovarian reserve stated by the serum levels of anti-Müllerian hormone (AMH) and follicle-stimulating hormone (FSH), and the antral follicles count (AF) in transvaginal ultrasonography; a normal anatomy of the uterus in transvaginal ultrasonography and saline contrast ultrasonohysterography; the absence of systemic autoimmune diseases; and signed informed consent. Only patients who had not previously been vaccinated against COVID-19 were included in the study. The exclusion criteria comprised: absolute contraindications for ART treatment, morbid obesity (BMI ≥ 40.0 kg/m^2^), HIV infection, and the implication of egg donation and surrogacy programs. Depending on their history of COVID-19, the patients were divided into two groups: group 1 included 105 patients who had no history of COVID-19 and group 2 included 135 patients who had experienced mild (n = 85) or moderate COVID-19 (n = 50) less than 12 months prior to oocyte retrieval.

The history of COVID-19 in the patients was confirmed by data from the Uniform State Health Information System of Russia, as well as via additional testing of their blood serum for SARS-CoV-2 antibodies. The severity of this COVID-19 was assessed in accordance with the Interim National Guideline “Prevention, diagnosis and treatment of novel coronavirus infection (COVID-19)” (version 13.1 of 17 November 2021). The diagnostic criteria for a mild form of COVID-19 were a positive result for SARS-CoV-2 using a real-time reverse-transcription polymerase chain reaction (RT-PCR) assay with a nasopharyngeal swab and the following clinical symptoms: a body temperature of < 38 °C, cough, weakness, a sore throat, and the absence of criteria for a severe or moderate infection. The criteria for the moderate form of the disease were: a body temperature above subfebrile (≥38 °C); shortness of breath during physical exertion with a respiratory rate of > 22 breaths per minute; changes in computed tomography (CT) or X-radiography that are typical of a viral infection; and SpO^2^ < 95%; serum C-reactive protein (CRP) > 10 mg/L.

In order to detect the re-infection of SARS-CoV-2, the patients were screened using an RT-PCR not earlier than 48 h before the transvaginal oocyte retrieval (TOR) and embryo transfer (ET).

To determine antibodies to SARS-CoV-2 in the blood serum, the “Kit of reagents for the detection of class G antibodies to the SARS-CoV-2 spike protein by enzyme immunoassay” (“DS-ELISA-ANTI-SARS-CoV-2-G (S)”; Diagnostic Systems LLC, Nijni Novgorod, Russia) was used. The result of the analysis was defined as the value of the positivity index (PI), calculated as the ratio of the optical density (OD) of the sample to the cut-off value. At a PI of >1.2, the result was considered to be positive, at a PI of <0.8 to be negative, and at a PI in the range of 0.8–1.2 to be equivocal.

Enzyme-linked immunosorbent assays (ELISA) were used for quantitative measurements of autoantibodies of different specificities in the blood serum of the patients. IgG and IgM antiphospholipid antibodies (aPL) to cardiolipin (CL), β2-glycoprotein-I (β2-GP-I), and annexin V (An V) and IgG antibodies to ds-DNA, thyroglobulin (TG), and thyroperoxidase (TPO) were defined using ORGENTEC Diagnostika GmbH kits (Mainz, Germany). IgG and IgM antibodies to phosphatidylethanolamine (PE) and the phosphatidylserine/prothrombin complex (PS/PT) were identified using AESKU.DIAGNOSTICS GmbH kits (Wendelsheim, Germany). The antibodies were determined according to the manufacturer’s instructions. The serum samples were tested at a dilution of 1:100. The optical density (OD) was measured at 450 nm using an automatic enzyme immunoassay analyzer Infinite F50 (TECAN, Grödig, Austria). To obtain quantitative results, a calibration curve for the dependence of the optical density on the concentration of calibrators was produced by a software making a 4-parameter linear-logarithmic approximation of the OD and analyte concentration values.

For the qualitative determination of IgG antibodies to 26 nuclear antigens using an ANA-Detect kit (ORGENTEC Diagnostika GmbH), the PI was calculated as the OD of the analyzed sample/OD of the control ratio.

Medizym® TRA kits (Medipan GmbH, Dahlewitz/Berlin, Germany) were used to detect antibodies to the thyroid-stimulating hormone receptor (TSHr) using a competitive ELISA with multiple consecutive incubations. The serum samples were tested undiluted using a TSHr-coated plate, biotinylated anti-TSHr antibodies, and a streptavidin-peroxidase conjugate. The absorption of the solution in the wells after the colorimetric reaction was measured at 450 nm on an automatic analyzer Infinite F50 (TECAN, Grödig, Austria).

A controlled ovarian hyperstimulation protocol with a gonadotropin-releasing hormone (GnRH) antagonist was used for the in vitro fertilization (IVF) or intracytoplasmic sperm injection (ICSI) treatment of the patients. All the patients received recombinant FSH or human menopausal gonadotropin (hMG), with individual dose adjustments. When the leading follicle reached 19 mm in diameter, 8000–10,000 IU of human chorionic gonadotropin (hCG) was administered as an ovulation trigger. TOR was performed under ultrasound guidance 36 h after the introduction of the ovulation trigger. Mature oocytes were fertilized using IVF or ICSI. Normal fertilization was stated when two symmetrical pronuclei were observed in the cytoplasm at 16–18 h. The zygotes were transferred to the COOK culture medium (COOK Medical, Bloomington, IN, USA) for further cultivation. A morphological assessment of the embryos was carried out after 120–122 h (on the 5th day) of cultivation using the Gardner classification [27]; afterwards, one or two best-quality embryos were transferred into the uterine cavity. Luteal support was provided with micronized progesterone (600 mg daily) or dydrogesterone (30 mg daily). Biochemical pregnancy was defined by the blood level of β-hCG 14 days after ET. Clinical pregnancy was stated 21 days after ET with ultrasound signs of intrauterine fetal sac. Live birth was defined as any delivery in which at least one live child was born at a gestational age of ≥ 24 weeks with a body weight of ≥ 500 g.

Data on the treatment outcome parameters (number of retrieved oocytes and MII-stage oocytes, fertilization rate, total number of blastocysts, blastulation rate, blastocyst grade, biochemical and clinical pregnancy rate, number of twin pregnancies, live birth rate, and spontaneous miscarriage rate) were recorded.

Statistical data processing was carried out using Microsoft Excel tables and the Statistica 10 software package (StatSoft Inc., Tulsa, OK, USA). To evaluate the qualitative data, proportions (%) were calculated. The χ^2^ test was used to compare the categorical data. The type of quantitative data distribution in the study groups was assessed using the Shapiro–Wilk test. Non-normally distributed data were presented as medians with an interquartile range (Me (Q25–Q75)) or minimum and maximum values (Me (min–max), and non-parametric statistical methods were used to compare two or three nonrelated groups, namely the Mann–Whitney U-test or Kruskal–Wallis test. The relationships between the variables were identified using Spearman’s rank correlation coefficient. The association between risk factors and outcomes was assessed using the relative risk (RR) with a 95% confidence interval (CI). Differences in the parameters between the groups were considered statistically significant at *p* ˂ 0.05.

## 3. Results

The study groups did not differ significantly in the mean age of the patients and the ratio of patients of early and late reproductive ages (Table 1).

The group 2 patients with a history of COVID-19 had a higher body mass index (BMI) and higher incidence of ear, nose, throat (ENT) and allergic diseases in comparison to the group 1 patients. There were no differences between groups 1 and 2 in the average number of pregnancies and childbirths, as well as in the incidence of gynecological diseases and recurrent miscarriages, except for adenomyosis, which was diagnosed more often in group 1 (*p* = 0.013). In group 2, 126 of 135 (93.3%) patients who had recovered from COVID-19 were positive for specific antiviral IgG antibodies, when tested 152 (91–243) days after the disease, and the median level of these antibodies in group 2 was significantly higher than that in group 1 (*p* < 0.0001) and did not differ between the patients with mild and moderate COVID-19 (*p* = 0.465). The frequency of gynecological and somatic diseases, the duration of infertility, and the number of ART cycles also did not differ between the patients with different forms of COVID-19.

The ICSI was used for oocyte fertilization in 91 of 105 patients (86.7%) in group 1 and in 124 of 135 patients (91.8%) in group 2; its frequency did not differ between groups 1 and 2 (*p* = 0.192). In other cases, IVF was used to fertilize eggs in both groups. The parameters of oogenesis and early embryogenesis, such as the number of total and mature (MII) oocytes, the fertilization rate, the number of zygotes and blastocysts obtained, and the proportion of blastocysts of excellent and poor quality, did not differ between groups 1 and 2 (Table 2). Moreover, similar parameters of oogenesis and embryogenesis in patients with mild and moderate COVID-19 also did not differ.

The time interval from COVID-19 to oocyte retrieval ranged from 35 days to 353 days, averaging 160 (80–249) days in the patients with mild COVID-19 and 148 (96–225) days in the patients with moderate COVID-19, not differing between these subgroups (*p* = 0.91). All post-COVID patients were divided into two subgroups depending on the time interval of ≤180 days or >180 days. Of all the parameters of oogenesis and embryogenesis, only the proportion of poor-quality blastocysts (category C) on the 5th day of cultivation was significantly higher in the patients with a time interval from COVID-19 to TOR of ≤180 days compared to the same parameter in the patients with a time interval of >180 days (*p* = 0.006) (Table 3).

The rates of biochemical and clinical pregnancy, twin pregnancy, and live birth did not differ between groups 1 and 2, as well as between subgroups 2a and 2b (Table 4). However, in patients with a history of moderate COVID-19, the incidence of miscarriage in the first trimester was higher than that in patients without a history of COVID-19 (*p* = 0.024), and the risk of miscarriage in subgroup 2b was 4.2 times higher compared to that in group 1 (RR = 4.2; 95% CI 1.09–16.11; *p* = 0.036).

An increase in the serum level of total aPL (IgM, IgG) over the reference values (RVs) was detected in 31/105 (29.5%) patients of group 1 and in 42/135 (31.1%) patients of group 2. The frequency of positive cases did not differ between groups 1 and 2 (*p* = 0.79). IgM and IgG antibodies to PE and IgG antibodies to An V were revealed in group 2 more often than antibodies to CL and β2-GP-I (*p*< 0.05). At the same time, IgG antibodies to PE and An V were detected in group 2 (*p* = 0.028; *p* = 0.035) and subgroup 2b (*p* = 0.024; *p* = 0.021) more often compared to group 1 (Table 5). The median levels of anti-β2-GP-I, anti-PS/PT IgG, and anti-An V IgM were higher in group 1, while the median levels of anti-PE IgG and anti-PS/PT IgM were higher in group 2 than in group 1 (Table 6).

ANAs were found only in a few patients in group 1 and in two out of three positive patients in combination with anti-dsDNA IgG, while in all patients of group 2, the levels of ANAs were within the reference values (Table 6). Anti-dsDNA antibodies were detected in both groups with similar rates (*p* = 0.45). Their levels also did not differ between groups 1 and 2 (Table 6).

The frequency of detection of antithyroid antibodies, in particular antibodies to TPO and TG, did not differ between groups 1 and 2 (*p* > 0.05). However, an increase in the level of anti-TSHr IgG was revealed more often in the post-COVID-19 patients than the patients without a history of COVID-19 (*p* = 0.033) (Table 5). The median levels of IgG antibodies to TSHr and TG were higher in group 2 compared to those in group 1 (Table 6).

The detection rate and level of tested autoantibodies of different specificities did not differ in patients with a history of mild or moderate COVID-19. It should be noted that, in the post-COVID-19 patients, all the tested autoantibodies did not correlate with the oogenesis and embryogenesis parameters and clinical outcomes, with the exception of the antibodies to PE. A weak negative correlation was found only between the level of anti-PE IgG and the number of obtained mature oocytes (r = −0.129, *p* = 0.045), as well as between the level of anti-PE IgG and the number of obtained zygotes (r = −0.132, *p* = 0.041). In addition, elevated serum levels of IgM antibodies to PE and An V were found in three out of six patients who had spontaneous miscarriages after the current ART cycle.

## 4. Discussion

In the present study, no differences were found between the parameters of oogenesis, embryogenesis, pregnancy, and live birth rates in the groups of patients with or without a history of COVID-19 (Table 2 and Table 4), which is consistent with the data of other researchers [28,29]. According to our results, these parameters also did not differ in patients with a history of mild or moderate COVID-19. It is assumed that a history of asymptomatic or mild SARS-CoV-2 infection in a female does not adversely affect the laboratory and clinical outcomes of fresh and frozen ET cycles [14]. However, it should be noted that, in the present study, despite the absence of differences in the rates of clinical pregnancy and live birth, the risk of early miscarriage in patients after moderate COVID-19 was 4.2 times higher compared to that in patients without a history of COVID-19 (Table 4).

Y. Herrero et al. showed a negative effect of SARS-CoV-2 on ovarian function, ovarian microvasculature, and folliculogenesis, in particular by altering the composition of the follicular fluid [30]. At the same time, the negative relationship found between the level of IgG to SARS-CoV-2 in the follicular fluid and the number of total and mature oocytes confirms that COVID-19 can adversely affect reproductive outcomes. Oocytes, embryos, and particularly late blastocysts seem to have the receptor/protease machinery to be susceptible to SARS-CoV-2 infection, although no viral RNA in oocytes has yet been found and data on embryos are conflicting [31].

A significantly lower proportion of high-quality embryos obtained in ART cycles after COVID-19 has been reported [17]. It is hypothesized that exposure to SARS-CoV-2 infection, which induces systemic inflammation, may impair the quality of the developing embryos. In another study, an observed slight decrease in the rate of blastocyst formation in the COVID-19 group may have been due to oxidative stress negatively affecting the quality of the oocytes [19], as well as the possible additional exposure of the virus to the developing embryo [13].

It is important to note that the comparison of the embryogenesis parameters between the large subgroups with time intervals from COVID-19 to TOR of ≤ 180 or >180 days, including patients with mild to moderate COVID-19, showed that the proportion of high-quality blastocysts (category A) did not differ between these subgroups (Table 3). However, the proportion of poor-quality blastocysts (category C) was higher in the patients who had COVID-19 ≤ 180 days before TOR. Apparently, this adverse outcome could be associated with a possible harmful impact of SARS-CoV-2 infection on oogenesis and oocyte quality. According to studies based on histological assessments of the ovaries, the entire duration of human folliculogenesis, from the primordial phase to the preovulatory phase, is estimated to be 175 days [32], which is consistent with our results. At the same time, Youngster et al. showed a negative effect of SARS-CoV-2 infection on oocyte yield when the time interval from COVID-19 to oocyte retrieval was more than 180 days; however, the number of patients in this subgroup was small [20].

It should be noticed that there are no data on the minimum required interval between COVID-19 recovery and ART treatment to ensure optimal outcomes. Orvieto R. et al. suggested the avoidance of ART treatment within the first 3 months after COVID-19 [17]. A confirmation of the need to delay ART treatment after COVID-19 and its duration of 3 or 6 months requires further clinical and laboratory studies.

It has been shown that COVID-19 can trigger autoimmune processes in genetically susceptible individuals [31]. The emergence of immune thrombocytopenic purpura, Guillain-Barré syndrome, and Miller-Fischer syndrome has been described in COVID-19 convalescents [33]. According to Sacchi M. C. et al., infection caused by SARS-CoV-2 is associated with the detection of autoantibodies of various specificities, including ANAs (in 57.5% of patients) and antibodies to CL (in 12.5%) and β2-GP-I (in 5% of patients) [34].

The present study demonstrated a high total detection rate of IgM and IgG aPL in the ART patients with or without a history of COVID-19. At the same time, an increase in the levels of antibodies to PE and An V was detected more often than in “criterial” aPL, in particular IgM and IgG antibodies to CL and β2-GP-I, which are currently the laboratory criteria for the diagnosis of APS. The post-COVID-19 patients more often had elevated levels of anti-PE and anti-An V IgG, as well as higher median levels of anti-PE IgG and anti-PS/PT IgM, compared to the patients without a history of COVID-19. These results are consistent with the data from a previous study demonstrating a low detection rate of “criterial” aPL and higher incidence of “non-criteria” aPL, in particular antibodies to An V and PT, in patients with COVID-19 [35]. The development of pro-inflammatory and pro-thrombotic conditions and the dysfunction and damage of vascular endothelial cells in COVID-19 lead to the exposure of negatively charged phospholipids, in particular PS, on the surface of activated and damaged cells, and the subsequent formation of complexes of phospholipids with phospholipid-binding proteins, such as An V and PT; this, in turn, promotes the production of autoantibodies to An V, PT, and PS/PT.

Many studies have investigated not only “criterial” aPL, but also “non-criteria” antibodies of a different specificity. According to a systematic review [36], such antibodies to the PS/PT complex were found in 0–24% of patients with COVID-19 and antibodies to An V were found in 3–19% of similar patients. It has been suggested that antibodies to PT and An V may be involved in the pathogenesis of COVID-19-associated coagulopathy, transiently increasing at a later stage of the disease or causing COVID-19-induced APS in susceptible patients and convalescents [35,37].

It is known that PE is widely present in human cell membranes and organelles, in particular mitochondria, and is unevenly distributed in the plasma membrane, mostly residing in its inner leaflet (>80%), playing an important role in cell division at the stage of cytokinesis [38]. Cell and tissue damage resulting from the infection and inflammation in COVID-19 may contribute to the displacement of PE to the outside and trigger the production of autoantibodies to PE. In addition, the production of autoantibodies can be facilitated by an increase in the content of PE in the infected cells and blood plasma in recovered patients with COVID-19, which is associated with the involvement of PE in the inhibition of the virus-induced inflammatory response [39].

The negative correlation between the level of anti-PE IgG and number of obtained mature oocytes and zygotes found in the present study is indirect evidence for the possibility of adverse effects of some aPL, persisting in the blood of the post-COVID-19 patients, on the outcomes of fresh oocyte ART cycles. Previously, Matsubayashi H. et al. showed that follicular fluid from aPL-seropositive patients was also positive for aPL of class G [40]. The presence of IgG aPL in the follicular fluid, in particular anti-PE IgG, was associated with a lower fertilization rate and, according to the authors, may be an independent risk factor for adverse outcomes of IVF cycles.

Since half of the patients with an early miscarriage had antibodies to PE and An V, it was suggested that autoimmune mechanisms could be involved in the development of this pregnancy complication. Previously, it has been shown that the antibodies to PE and An V are possible risk factors for a recurrent miscarriage [41,42]. These results are consistent with a meta-analysis that showed a higher incidence of aPL in women with failed IVF/ICSI cycles than women with successful cycles [43]. According to the same meta-analysis, aPL-positive women have a higher miscarriage rate than aPL-negative women; however, the rates of live birth and biochemical and clinical pregnancy in these women did not differ.

ANAs and anti-dsDNA antibodies in both groups were detected significantly less often than aPL. Actually, in the post-COVID-19 patients, ANAs were not detected, while anti-dsDNA antibodies were found in single patients of both groups. The mean contents of ANAs and anti-dsDNA antibodies did not differ between the two groups. Moreover, no correlation was found between the level of these antibodies and the outcomes of the ART cycles, which could have been due to the small number of seropositive cases.

However, according to a systematic review and meta-analysis (2019), the presence of ANAs can correlate with poor outcomes of IVF/ICSI cycles, such as low implantation and clinical pregnancy rates and high miscarriage rates. In addition, it has been shown that ANAs can adversely affect oocyte quality and embryonic development, leading to infertility and recurrent implantation failure [44,45]. Wu S. et al. recently showed that, in ART patients positive for ANAs in the follicular fluid, the number of retrieved oocytes, MII oocytes, fertilization, implantation, and clinical pregnancy rates were lower, while the early miscarriage rate was higher compared to the control group [46]. According to Fan J. et al., serum anti-dsDNA antibodies may be an important marker of defective oocytes or embryos and reduced rates of fertilization, implantation, and clinical pregnancy in infertile women with ANAs [47].

The patients after COVID-19 had significantly higher median levels of antibodies to thyroid antigens, such as TSHr and TG, and a higher incidence of anti-TSHr antibodies. Increased levels of antibodies to thyroid antigens (TPO and TG) have also been demonstrated 3 months after COVID-19 in Chinese patients [48]. Since COVID-19 can cause autoimmune damage to the thyroid gland, the further monitoring of convalescents with thyroid dysfunction is recommended after severe COVID-19.

According to a systematic review (2016), in women with biochemically normal thyroid function who underwent ART programs, anti-thyroid antibodies were associated with a significantly decreased chance of live birth and an increased risk of spontaneous miscarriage, but did not affect the number of oocytes retrieved and the frequency of the fertilization, implantation, and clinical pregnancy rates [49].

It should be noted that, in the present study, in the post-COVID-19 patients, the levels of antibodies to TPO, TG, and TSHr also did not correlate with the parameters of oogenesis and embryogenesis. At the same time, these patients had a higher incidence and mean level of anti-TSHr antibodies compared to the patients without a history of COVID-19. It is assumed that the possible blocking effect of these antibodies on the luteinizing hormone/hCG receptors in the corpus luteum, due to cross-reactivity between TSH, gonadotropins, and their receptors, can lead to a decrease in the production of steroid hormones and the threat of abortion [50].

## 5. Conclusions

Thus, in non-vaccinated patients who survived mild or moderate COVID-19 less than 12 months before oocyte retrieval, the parameters of oogenesis and embryogenesis and rates of biochemical and clinical pregnancy in fresh oocyte ART cycles did not differ from those in non-vaccinated patients without a history of COVID-19. However, patients surviving COVID-19 less than 180 days prior to oocyte retrieval had an increased proportion of poor-quality blastocysts and there was an increased rate of early miscarriage in patients after moderate COVID-19. In the post-COVID-19 patients, there was an increase in the levels of antiphospholipid antibodies, mainly “non-criteria” antibodies to PE and An V, as well as antibodies to thyroid antigens, and an inverse correlation of the serum antibodies to PE with the number of oocytes and zygotes obtained. All of the above suggests a possible adverse effect of COVID-19 and its associated autoantibodies on the outcomes of ART cycles with fresh oocytes and on early pregnancy.

The study has limitations such as an absence of measurements of the levels of serum autoantibodies in the patients with history of COVID-19 before the disease. Furthermore, our results cannot be extrapolated to patients without infertility. It should not be forgotten that women with infertility can have increased levels of serum autoantibodies, regardless of a history of COVID-19. Moreover, not all the endogenous mechanisms triggering autoantibody production and not all confounders were taken into account in the study. We believe that further dynamic monitoring of the clinical and laboratory parameters of patients with increased levels of serum autoantibodies is required to refine the data obtained.

## Figures and Tables

**Table 1:** Demographic, clinical, and laboratory characteristics of patients with or without a history of COVID-19.

Parameter	Non-COVID-19 (Group 1, n = 105)	COVID-19 (Group 2, n = 135)		*p*-Value
		Mild (Subgroup 2a, n = 85)	Moderate (Subgroup 2b, n = 50)	
Patient age, years	34 (30–36)	34 (31–37)		0.396 *
		35 (32–37)	33 (30–36)	0.088 *
Patient age > 35 years	31 (29.5%)	48(35.6%)		0.320 **
		33 (38.8%)	15 (30.0%)	0.298 **
BMI, kg/m^2^	21.9 (20.0–24.5)	22.9 (20.4–25.5)		0.009 *
		22.4 (20.1–25.3)	23.4 (21.2–26.4)	0.003 *
IgG to the SARS-CoV-2 spike protein, PI	0.13 (0.12–0.96)	6.08 (2.80–10.79)		<0.0001 *
		6.06 (2.88–10.53)	6.39 (2.80–11.43)	<0.0001 *
Gravidity	0 (0–6)	0 (0–5)		0.752 ***
		0 (0–5)	0 (0–5)	0.656 ***
Parity	0 (0–2)	0 (0–3)		0.992 **
		0 (0–3)	0 (0–3)	0.988 **
Recurrent abortion	3 (2.8%)	10 (7.4%)		0.122 **
		8 (9.4%)	2 (4%)	0.123 **
Endometriosis	25 (23.8%)	38 (28.1%)		0.448 **
		27 (31.7%)	11 (22%)	0.345 **
Adenomyosis	14 (13.3%)	6 (4.4%)		0.013 **
		3 (3.5%)	3 (6%)	0.041 **
Uterine fibroids	21 (20%)	33 (24.4%)		0.413 **
		25 (29.4%)	8 (16%)	0.141 **
Chronic endometritis	11 (10.5%)	8 (5.6%)		0.195 **
		5 (5.9%)	3 (6%)	0.432 **
Chronic salpingoophoritis	13 (12.4%)	15 (11.1%)		0.761 **
		8 (9.4%)	7 (14%)	0.692 **
Primary infertility	61 (58.1%)	79 (58.5%)		0.947 **
		49 (57.6%)	30 (60%)	0.962 **
Secondary infertility	44 (41.9%)	56 (41.5%)		0.95 **
		36 (42.4%)	20 (40%)	0.963 **
Duration of infertility, years	4 (3–6.5)	5 (3–6)		0.631**
		5 (3–6)	5 (2–6)	0.839 **
Number of ART cycles	1 (1–5)	1 (1–8)		0.370 ***
		1 (1–8)	1 (1–4)	0.429 ***
ENT diseases	9 (8.6%)	24 (17.8%)		0.039 **
		15 (17.6%)	9 (18%)	0.120 **
Endocrine diseases	31 (29.5%)	27 (20%)		0.087 **
		17 (20%)	10 (20%)	0.231 **
Allergic diseases	9 (8.6%)	23 (17%)		0.055 **
		11 (12.9%)	12 (24%)	0.030 **

* Me (Q 25–Q 75), Mann–Whitney U-test or Kruskal–Wallis test. ** Abs. (%), χ^2^-test. *** Me (min–max), Mann–Whitney U-test or Kruskal–Wallis test. BMI—body mass index; PI—positivity index; ART—assisted reproductive technology; and ENT—ear, nose, throat.

**Table 2:** Parameters of oogenesis and embryogenesis in fresh oocyte ART cycles.

Parameter	Non-COVID-19 (Group 1, n = 105)	COVID-19 (Group 2, n = 135)		*p*-Value
		Mild (Subgroup 2a, n = 85)	Moderate (Subgroup 2b, n = 50)	
Total oocytes *	10 (6–13)	9 (6–14)		0.366
		8 (5–14)	10 (6–16)	0.334
MII stage oocytes *	8 (5–11)	7 (4–11)		0.262
		7 (4–10)	7 (5–12)	0.367
MII stage oocytes/total oocytes **	83 (70–100)%	82 (70–95)%		0.518
		82 (71–100)%	80 (70–87)%	0.265
Fertilization *	6 (4–9)	6 (4–10)		0.375
		6 (4–9)	6 (4–10)	0.443
Fertilization rate **	90 (75–100)%	92 (80–100)%		0.391
		90 (80–100)%	100 (83–100)%	0.501
Total blastocysts *	3 (1–5)	3 (1–5)		0.324
		3 (1–5)	3 (1–6)	0.513
Blastulation rate **	50 (33–66)%	50 (25–71)%		0.980
		50 (25–75)%	54 (30–66)%	0.948
Blastocyst grade **				
A	72 (68.6%)	85 (63%)		0.37
		53 (62.4%)	32 (64%)	0.651
B	10 (9.5%)	10 (7.4%)		0.56
		8 (9.4%)	2 (4%)	0.46
C	11 (10.5%)	18 (13.3%)		0.51
		11 (12.9%)	7 (14%)	0.784
Grade A blastocysts *	1 (0–3)	1 (0–2)		0.188
		1 (0–2)	1 (0–2)	0.235
Grade A blastocysts/total blastocysts **	40 (0–67)%	33 (0–67)%		0.336
		33 (0–67)%	33 (0–67)%	0.572
Grade C blastocysts *	1 (0–2)	1 (0–2)		0.994
		1 (0–2)	1 (0–2)	0.999
Grade C blastocysts/total blastocysts **	33 (0–50)%	33 (0–67)%		0.468
		33 (0–67)%	29 (0–55)%	0.530

* Me (Q 25–Q 75), Mann–Whitney U-test or Kruskal–Wallis test. ** Abs. (%), χ^2^-test.

**Table 3:** Parameters of oogenesis and embryogenesis in patients with different time intervals from COVID-19 to oocyte retrieval.

Parameter	Time Interval ≤180 Days (n = 85)	Time Interval >180 Days (n = 50)	*p*-Value
Total oocytes *	8 (6–15)	9.5 (6–11)	0.749
MII stage oocytes *	7 (5–11)	6.5 (4–9)	0.338
MII stage oocytes/total oocytes **	83 (71–92)%	75 (60–100)%	0.249
Fertilization rate **	100 (80–100)%	90 (77–100)%	0.349
Total blastocysts *	3 (1–5)	3 (1–5)	0.456
Blastulation rate **	54 (30–71)%	50 (25–68)%	0.655
Grade A blastocysts *	1 (0–2)	1 (0–2)	0.665
Grade A blastocysts/total blastocysts **	33 (0–60)%	32 (0–66)%	0.998
Grade C blastocysts *	1 (1–2)	1 (0–2)	0.075
Grade C blastocysts/total blastocysts **	37 (14–71)%	18 (0–40)%	0.006

* Me (Q 25–Q 75), Mann–Whitney U-test, ** %, χ^2^-test.

**Table 4:** Outcomes of fresh oocyte ART cycles in patients with or without a history of COVID-19.

Parameter	Non-COVID-19 (Group 1, n = 105)	COVID-19 (Group 2, n = 135)		*p*-Value
		Mild (Subgroup 2a, n = 85)	Moderate (Subgroup 2b, n = 50)	
Biochemical pregnancy	32 (30.5%)	39 (28.9%)		0.789
		22 (25.8%)	17 (34%)	0.586
Clinical pregnancy	30 (28.6%)	39 (28.9%)		0.957
		22 (25.8%)	17 (34%)	0.602
Twin pregnancy	0	4 (2.9%)		0.134
		2 (2.3%)	2 (4%)	0.158
Childbirth	27 (25.7%)	30 (22.2%)		0.528
		19 (22.3%)	11 (22%)	0.217
Spontaneous miscarriage	3 (2.9%)	9 (6.7%)		0.179
		3 (3.5%)	6 (12%)	0.037 0.792 (1 vs. 2) 0.024 (1 vs. 3) 0.056 (2 vs. 3)
Miscarriages/clinical pregnancies	10%	23.1%		0.18
		13.6%	35.3%	0.038

Abs. (%), χ^2^ test.

**Table 5:** Detection rates of serum autoantibodies of different specificity.

Parameter	Reference Values	Non-COVID-19 (Group 1, n = 105)	COVID-19 (Group 2, n = 135)		*p*-Value
			Mild (Subgroup 2a, n = 85)	Moderate (Subgroup 2b, n = 50)	
anti-CL IgM	<7 MPL-U/mL	3 (2.9%)	4 (2.9%)		0.96
			3 (5.2%)	1 (2.0%)	0.877
anti-CL IgG	<10 GPL-U/mL	0 (0.0%)	0 (0.0%)		-
			0 (0.0%)	0 (0.0%)	-
anti-β2-GP-I IgM	<8 U/mL	1 (0.95%)	2 (1.5%)		0.75
			1 (1.2%)	1 (2.0%)	0.858
anti-β2-GP-I IgG	<8 U/mL	1 (0.95%)	2 (1.5%)		0.75
			1 (1.2%)	1 (2.0%)	0.858
anti-AnV IgM	<8 U/mL	4 (3.8%)	2 (1.5%)		0.259
			1 (1.2%)	1 (2.0%)	0.496
anti-AnV IgG	<8 U/mL	2 (1.9%)	11 (8.1%)		0.035
			6 (7.1%)	5 (10%)	0.081
anti-PE IgM	<18 U/mL	21 (20%)	25 (18.5%)		0.55
			13 (15.3%)	12 (24%)	0.444
anti-PE IgG	<18 U/mL	1 (0.95%)	9 (6.7%)		0.028
			5 (5.9%)	4 (8%)	0.075
anti-PS/PT IgM	<18 U/mL	3 (2.9%)	2 (1.5%)		0.455
			2 (2.4%)	0 (0.0%)	0.496
anti-PS/PT IgG	<18 U/mL	4 (3.8%)	4 (2.9%)		0.699
			4 (4.7%)	0 (0.0%)	0.317
ANA (IgG)	<1.2 PI	3 (2.9%)	0 (0.0%)		0.047
			0 (0.0%)	0 (0.0%)	0.142
anti-dsDNA IgG	<20 IU/mL	8 (7.6%)	7 (5.2%)		0.441
			3 (5.2%)	4 (8%)	0.434
anti-TPO IgG	<50 IU/mL	5 (4.7%)	10 (7.4%)		0.401
			6 (7.1%)	4 (8%)	0.686
anti-TSHr IgG	<1.5 IU/mL	2 (1.9%)	11 (8.2%)		0.033
			9 (10.6%)	2 (4%)	0.028
anti-TG IgG	<100 IU/mL	4 (3.8%)	8 (5.9%)		0.455
			5 (5.9%)	3 (6%)	0.757

Abs. (%), χ^2^ test. CL—cardiolipin; β2-GP-I—β2-glycoprotein-I; An V—annexin V; PE—phosphatidylethanolamine; PS/PT—phosphatidylserine/prothrombin; ANA—antinuclear antibodies; dsDNA—double-stranded DNA; TPO—thyroperoxidase; TSHr—thyroid-stimulating hormone receptor; and TG—thyroglobulin.

**Table 6:** Serum level of autoantibodies of different specificity.

Parameter	Reference Values	Non-COVID-19 (Group 1, n = 105)	COVID-19 (Group 2, n = 135)		*p*-Value
			Mild (Subgroup 2a, n = 85)	Moderate (Subgroup 2b, n = 50)	
anti-CL IgM	<7 MPL-U/mL	3.03 (1.94–4.05)	2.52 (1.59–3.91)		0.137 *
			2.43 (1.59–1.04)	3.04 (1.50–3.8)	0.303 **
anti-CL IgG	<10 GPL-U/mL	1.87 (1.41–2.56)	2.10 (1.59–3.01)		0.063
			2.01 (1.50–2.86)	2.14 (1.68–3.31)	0.083
anti-β2-GP-I IgM	<8 U/mL	1.51 (0.81–2.43)	1.41 (0.98–2.17)		0.871
			1.41 (0.95–2.38)	1.42 (1.06–2.07)	0.957
anti-β2-GP-I IgG	<8 U/mL	2.98 (2.12–3.59)	2.37 (1.21–3.26)		0.001
			2.09 (0.94–2.30)	2.52 (1.94–3.54)	0.0004
anti-AnV IgM	<8 U/mL	2.52 (1.76–3.52)	2.22 (1.23–3.22)		0.030
			2.22 (1.26–3.18)	2.25 (1.45–3.35)	0.068
anti-AnV IgG	<8 U/mL	2.91 (2.27–3.94)	3.34 (2.13–4.60)		0.238
			3.37 (2.20–4.95)	3.23 (2.00–4.58)	0.253
anti-PE IgM	<18 U/mL	12.23 (8.70–16.98)	11.85 (8.67–15.58)		0.544
			11.93 (7.78–15.2)	11.61 (9.09–7.89)	0.513
anti-PE IgG	<18 U/mL	6.57 (5.78–7.77)	7.82 (6.25–9.74)		0.0002
			7.39 (6.20–8.89)	8.20 (6.74–10.93)	0.0027
anti-PS/PT IgM	<18 U/mL	1.72 (1.10–3.28)	2.39 (1.47–3.58)		0.009
			2.39 (1.53–3.73)	2.33 (1.28–3.55)	0.027
anti-PS/PT IgG	<18 U/mL	4.24 (3.00–5.36)	3.38 (2.28–5.31)		0.027
			3.43 (2.32–5.48)	3.02 (2.24–5.11)	0.057
ANA (IgG)	<1.2 PI	0.30 (0.30–0.40)	0.30 (0.30–0.40)		0.505
			0.40 (0.30–0.40)	0.30 (0.30–0.40)	0.626
anti-dsDNA IgG	<20 IU/mL	13.65 (10.24–16.77)	13.34 (10.34–6.23)		0.531
			12.33 (10.30–15.75)	14.06 (10.42–17.23)	0.467
anti-TPO IgG	<50 IU/mL	12.04 (5.31–16.44)	14.35 (5.91–20.07)		0.064
			14.35 (6.58–20.54)	14.51 (5.26–19.26)	0.162
anti-TSHr IgG	<1.5 IU/mL	0.49 (0.28–0.96)	0.76 (0.41–1.16)		0.002
			0.83 (0.43–1.21)	0.68 (0.37–1.02)	0.004
anti-TG IgG	<100 IU/mL	9.13 (4.13–27.22)	18.18 (6.84–36.98)		0.008
			20.6 (6.29–35.81)	17.85 (7.97–37.67)	0.026

Me (Q 25–Q 75), *—Mann-Whitney U-test, **—Kruskal-Wallis test. CL—cardiolipin; β2-GP-I—β2-glycoprotein-I; An V—annexin V; PE—phosphatidylethanolamine; PS/PT—phosphatidylserine/prothrombin; ANA—antinuclear antibodies; dsDNA—double-stranded DNA; TPO—thyroperoxidase; TSHr—thyroid-stimulating hormone receptor; and TG—thyroglobulin.

## Data Availability

The data presented in this study are available on request from the corresponding author. The data are not publicly available due to privacy or ethical restrictions.
